# Higher Cardiorespiratory Fitness Levels May Attenuate the Detrimental Association between Weight Status, Metabolic Phenotype and C-Reactive Protein in Adolescents—A Multi-Cohort Study

**DOI:** 10.3390/nu12051461

**Published:** 2020-05-18

**Authors:** Cesar Agostinis-Sobrinho, Rafaela Rosário, Rute Santos, Sigute Norkiene, Jorge Mota, Alona Rauckienė-Michaelsson, Katherine González-Ruíz, Mikel Izquierdo, Antonio Garcia-Hermoso, Robinson Ramírez-Vélez

**Affiliations:** 1 Faculty of Health and Sciences, Klaipeda University, 92294 Klaipeda, Lithuania; cesaragostinis@hotmail.com (C.A.-S.); sigute.norkiene@jurlig.lt (S.N.); alona.rauckiene@gmail.com (A.R.-M.); 2 School of Nursing, University of Minho, 4710 Braga, Portugal; rrosario@ese.uminho.pt; 3 Health Sciences Research Unit: Nursing (UICISA: E), Nursing School of Coimbra (ESEnfC), 3000-232 Coimbra, Portugal; 4 Research Centre in Physical Activity, Health and Leisure (CIAFEL), Faculty of Sport, University of Porto, 4200-450 Porto, Portugal; rutemarinasantos@hotmail.com (R.S.); jmota@fade.up.pt (J.M.); 5 Grupo de Ejercicio Físico y Deportes, Vicerrectoría de Investigaciones, Universidad Manuela Beltrán, Bogotá 110231, Colombia; katherine.gonzalez@docentes.umb.edu.co; 6 Department of Health Sciences, Public University of Navarra, Navarrabiomed-IdiSNA, Complejo Hospitalario de Navarra (CHN), 31008 Pamplona, Spain; mikel.izquierdo@gmail.com (M.I.); antonio.garcia.h@usach.cl (A.G.-H.); 7 CIBER of Frailty and Healthy Aging (CIBERFES), Instituto de Salud Carlos III, 28029 Madrid, Spain; 8 Laboratorio de Ciencias de la Actividad Física, el Deporte y la Salud, Facultad de Ciencias Médicas, Universidad de Santiago de Chile, USACH, Santiago 7500618, Chile

**Keywords:** metabolic, healthy, obese, aerobic, fitness, inflammation, youth

## Abstract

Results from several studies show that only obese, unfit subjects, but not obese, fit subjects, are at higher mortality risk than are normal-weight fit subjects. The aim of the study was two-fold: (1) to examine the differences in C-reactive protein levels across different metabolic phenotypes (healthy and unhealthy) of weight status and (2) ascertain whether high levels of cardiorespiratory fitness (CRF) attenuate the association of C-reactive protein and metabolic phenotypes of weight status. This was a pooled study, which included data from three cross-sectional projects (1706 youth (921 girls) aged 12–18 years). We used a Shuttle run test to assess CRF. Adolescents were classified into six metabolic phenotypes (healthy and unhealthy) of weight status (non-overweight, overweight and obese), based on age- and sex-specific cutoff points for triglycerides, systolic blood pressure, HDL-cholesterol, glucose and body mass index. High-sensitivity assays were used to obtain the C-reactive protein as inflammatory biomarker. After adjustment for potential confounders (age, sex, pubertal stage and country), the analysis of covariance (ANCOVA) shows that C-reactive protein is directly associated with metabolic phenotypes of weight status. Subjects with obesity, regardless of their metabolic profile, had higher levels of C-reactive protein Z-score. In addition, (after adjustments for potential confounders) a two-way ANCOVA showed that high levels of CRF attenuated the associations of C-reactive protein levels in metabolic healthy non-overweight and in adolescents with obesity. In conclusion, higher CRF levels may attenuate the detrimental association between obesity and C-reactive protein independently of metabolic phenotype. Findings from this study are important for prevention, clinical practice on issues associated with adiposity and metabolic disorders.

## 1. Introduction

Obesity is characterized by an excessive accumulation of adipose tissue, and this condition is generally associated with cardiometabolic risk and low-grade inflammation. However, accumulated evidence has shown that some obese subjects could have a healthy metabolic profile (metabolically health but obese—MHOB), and subjects with normal weight could present an abnormal metabolic profile (metabolically unhealthy but normal weight MUNO) [1]. Those with MHOB are a subset of subjects who have a BMI cutoff point for obesity (≥30 kg/m^2^), but do not have other major cardiovascular risk factors [1]. There is growing evidence suggesting that low-grade inflammation may be the fundamental factor that determines metabolic differences between subgroups of obesity [1,2].

Chronic inflammation has a key role as a pathogenic mechanism in the origin and development of cardiovascular diseases (CVD) [3]. Of the wide array of inflammatory biomarkers available, high-sensitivity C-reactive protein is one of the most commonly used in clinical settings for access inflammatory levels [4]. C-reactive protein (CRP) is an acute inflammatory protein, produced predominantly in the liver, in response to several cytokines, which induce endothelial dysfunction, accelerate progression of atherosclerosis and increase the risk of cardiovascular disease [5]. In addition, CRP is also associated with several other inflammatory markers, including leukocyte count, fibrinogen, albumin, and erythrocyte sedimentation rate [5]. CRP is believed to be also both a stronger marker of subclinical inflammation and a useful predictor of stroke, coronary heart disease and mortality risk [4]. Thus, despite some controversies surrounding the measurement of this biomarker [5], CRP has been linked to early screening of CVD and Diabetes Mellitus type 2, not only in adults [4], but also in adolescents [6].

In the same line, the assessment of fitness during adolescence has called for a better understanding of the interrelationship between body composition and health-related physical fitness in youth [7]. Similarly, previously epidemiologic studies have shown that youths with low body fat and low cardiorespiratory fitness (CRF) were at high cardiovascular risk than those with high body fat and adequate CRF [8,9]. Excessive body fat and low CRF are associated with a list of severe diseases, including metabolic disorders [10,11], independently of weight status or dietary intake [12]; however, it is not entirely clear if the interrelationships among CRF, body composition and low-grade inflammation in adolescents are independent or partly due to the mediating effect of excessive adiposity, since the current literature is limited and/or equivocal [13,14]. Moreover, the effects of CRF combined with body composition on subclinical inflammation during the adolescence are unclear. 

Results from several studies show that only obese, unfit (low cardiorespiratory fitness as determined by maximal exercise testing), but not obese and fit subjects, are at higher mortality risk than normal-weight fit subjects [1]. Based on this, it is possible that optimal CRF levels will be related to a decrease in body composition and subclinical inflammation, leading to a healthy metabolic profile, despite the presence of high body fat, referred to as the MHOB phenotype. Therefore, we hypothesized that CRF is negatively related to metabolic unhealthy phenotypes and inflammation independently of sex and age. With this study, we aimed to (1) examine the differences in CRP levels across different metabolic phenotypes (unhealthy and healthy in non-overweight, overweight and obese) and (2) ascertain whether high levels of CRF attenuate the association of CRP and metabolic phenotypes of weight status. This approach will help us to better understand the role and the underling effect of CRF on inflammation, as well as present the novelty of analyzing the data according to different metabolic phenotypes in a multi-cohort study and relatively large sample size.

## 2. Materials and Methods

### 2.1. Design and Study Population

We pooled data from three studies, Fuprecol study [15], the LabMed Physical Activity Study [6,16] and the Azorean Physical Activity and Health Study II [17], for the purpose of increasing sample size of metabolic subgroups of overweight and obese. Detailed descriptions of sampling and recruitment approaches and data collection are described elsewhere [6,16,18,19]. In brief, The Fuprecol Study was approved by the Ethical Committee of the Rosario University Board (Code CEI-ABN026-000262). Participants aged 12–17.9 years were recruited from a non-random selection of 20 public schools (grades 5 through 11) from Bogotá-Colombia. Blood sampling was randomly performed in one-third of the recruited subjects. The Azorean Physical Activity and Health Study II was approved by the faculty and the Portuguese Foundation for Science and Technology ethics committee. Participants aged 14–18 years were recruited by means of a proportionate stratified random sampling, taking into account the location (island) and the number of adolescents by age and sex in each school, where 95% of the population lives (six of the nine Azorean Islands—Portugal: S. Jorge, Graciosa, S. Miguel, Terceira, Faial, and Pico). The LabMed Physical Activity Study was approved by the Ethics Committee of the Faculty of Sport, at the University of Porto. Participants aged 12–18 years were from four Portuguese cities (Barcelos, Braga, Vila Nova de Gaia and Ílhavo). The schools were selected based on pragmatic, budgetary and logistical reasons. All the studies were conducted in accordance with the Helsinki Declaration for Human Studies, and informed consents were signed by participants and their parents or legal guardians. For this study, which only included youths with completed data, (total= 1706 adolescents (57.3 girls)) the same methodologies and approaches for all variables were used. The number of subjects included from each study was as follow; the Fuprecol study total N = 660 (357 girls), the LabMed Physical Activity Study total = 529 (267 girls), the Azorean Physical Activity and Health Study II (total = 517 (297 girls). All participants were asked to not perform any vigorous physical activity during the 48 h prior to any clinical evaluation.

### 2.2. Anthropometrics

Weight and standing height were measured, respectively, with a stadiometer (Harpenden Stadiometer Holtain Ltd., Crymmych, Pembrokeshire, UK, and Seca 213–217, Hamburg, Germany) and an portable electronic weight scale (Tanita Inner Scan model BC 532 and Tanita model BF689 Tokyo, Japan). Body mass index (BMI) was calculated as the ratio of body weight (kg) to body standing height (kg/m^2^). 

### 2.3. Cardiorespiratory Fitness (CRF)

Assessment of CRF was done by the 20 m Shuttle Run Test (20 m SRT), as described previously [20]. From laps performed and using the equation reported by Leger et al. [20], we estimated the peak oxygen consumption (VO_2_ peak, mL/kg/min). The adolescents were classified in two groups (low CRF and high CRF), according to the proposed cutoff for this population by Ruiz et al. [21]. 

### 2.4. Cardiometabolic Outcomes

Blood samples were collected (for Cholesterol linked to high-density lipoproteins (HDL-c), serum triglycerides (TG), fasting glucose and high-sensitive CRP after an overnight fast, according to standardized procedures, as follows: For the Fuprecol study, high-sensitive C-reactive protein was obtained, using the turbidimetric method with QuikRead 101 equipment (Both Orion Diagnostic). Cardiocheck^®^ equipment (Mexglobal SA, Parsippany, NJ, USA) was used to determine concentrations of fasting glucose, TG and HDL-c. For the Azorean Physical Activity and Health Study II, high-sensitive C-reactive protein was measured by enzyme-linked immunosorbent assay (IMMULITE 2000, SIEMENS, 2000, Diagnostic Products Corporation, Los Angeles, CA, USA). Fasting glucose, TG and HDL-c were measured on a Cobas Integra 400 Plus, by colorimetric methods (ROCHE 227 Diagnostics, Indianapolis, IN, USA). The LabMed study used high-sensitive CRP, latex-enhanced immunoturbidimetric assay (Siemens Advia 1600/1800) and measured HDL cholesterol by precipitation of the apolipoprotein B containing lipoproteins with dextran-magnesium-chloride method (Advia1600/1800, Siemens, Erlangen, Germany); fasting glucose by hexokinase method (Siemens Advia 1600/1800, Erlangen, Germany); and TG levels by enzyme glycerol phosphate oxidase method (Siemens Advia 1600/1800, Erlangen, Germany). Finally, blood pressure determination was made after the participants had been sitting for at least 5 min, with an automated blood pressure monitor (Omrom^®^ HEM 705 CP/Omron, Healthcare, Kyoto, Japan and model BP 8800, Critikon, Inc., Tampa, FL, USA).

### 2.5. Pubertal Stage

All the adolescents self-assessed their pubertal stage (ranging from stage I to V for boys and girls separately) of secondary sex characteristics based on the Tanner staging system [22]; for girls, images are provided of the five stages of development of both pubic hair and breasts, and for boys, images shows the five stages of pubic hair development and illustrations of testicular volume. 

### 2.6. Definition of Metabolically Healthy and Metabolically Unhealthy Status

Metabolically (un)healthy individuals were defined as having 0 (for normal values) or 1 (for abnormal values) of the following risk factors: TG, glucose, HDL-c and diastolic or systolic blood pressure. For this, we used age- and sex-specific cut-points for the metabolic phenotype classification that has been published for adults [23] (linked to—IDF—International Diabetes Federation, revised and modified by Cook) and adapted for youths [24]. If the children presented two or more unhealthy metabolic risk factors, they were considered to have a metabolic unhealthy phenotype. These approaches have been used recently in several studies [25,26,27]. Weight status (BMI) was defined according to the criteria of the WHO [28]. As a criterion, and in agreement with previous studies [25,29,30], we excluded waist circumference, since most of the individuals with higher weight status have a waist circumference above the specific thresholds of age and sex for the metabolic syndrome [23,31]. Thus, six metabolic phenotypes were created according to BMI (non-overweight, overweight and obese) and for (healthy and unhealthy metabolic phenotypes): metabolically healthy non-overweight (MHNO); metabolically unhealthy non-overweight (MUNO); metabolically healthy overweight (MHOV); metabolically unhealthy overweight (MUOV); metabolically healthy obese (MHOB); and metabolically unhealthy obese (MUOB).

### 2.7. Statistical Analysis

Data was analyzed by using the SPSS for Windows version 26.0 (SPSS Inc., Chicago, IL, USA). Descriptive data are presented as means and standard deviation, unless otherwise stated. The assumptions of normality and data distribution were assessed by the Kolmogorov–Smirnov test. Because of a non-normal distribution, high-sensitive CRP values were standardized prior to the analyses (Z-score = (participant’s value - mean value of the sample)/standard deviation). Interaction between our independent variable and databases was tested, and no significant interaction was observed. The one-way ANCOVA (analysis of covariance) with Bonferroni correction adjusted for age, sex, pubertal stage and country were used to assess the differences between mean values of high-sensitive CRP z-scores across six metabolic phenotypes of weight status. Differences in the high-sensitive CRP according to metabolic phenotypes’ categories and CRF status were analyzed, using a two-way ANCOVA to obtain adjusted means. Additionally, the one-way ANCOVA was used to test the influence of CRF status within each metabolic phenotype ((un)healthy in individuals with normal weight, overweight and obesity) adjusted for age, sex, pubertal stage and country. Bonferroni post hoc multiple comparison tests were used to assess the differences between Fit and Unfit. A polynomial linear test was used to assess linear trends (version 25.0 SPSS Inc., Chicago, IL, with a level of significance of 0.05). 

## 3. Results

Descriptive characteristics of the participants, according to weight status and metabolic health status, are presented in Table 1. In total, 1706 adolescents participated in the study, 30.4% of whom were considered overweight and obese. In each category of weight status, the metabolically unhealthy profile had significantly higher mean levels of cardiovascular risks of triglycerides, glucose, SBP and DBP.

**Figure 1:**
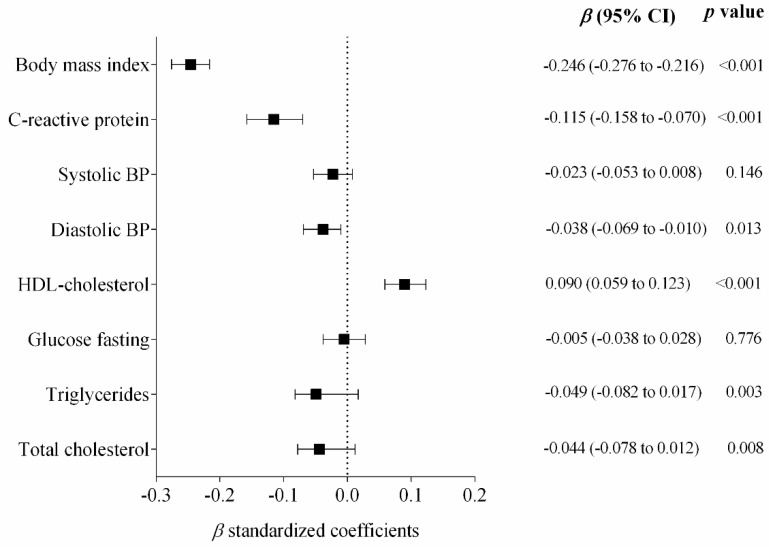
Standardized regression coefficients examining the association of cardiorespiratory fitness and cardiometabolic markers. Cardiorespiratory Fitness (Independent variable). Bars represent adjusted means and 95% confidence intervals, adjusted for age, sex, pubertal stage and country.

The regression analyses (Figure 1) showed a significant inverse association between cardiorespiratory fitness and BMI (standardized β = −0.246; *p* < 0.001), high-sensitive CRP (standardized β = −0.115; *p* < 0.001), Diastolic BP (standardized β = −0.038; *p* = 0.013), Triglycerides (standardized β = −0.049; *p* = 0.003) and total cholesterol (standardized β = −0.044; *p* = 0.008), as well as a direct association with HDL-cholesterol (standardized β = 0.090; *p* < 0.001), after adjustments for age, sex, pubertal stage and country.

**Figure 2:**
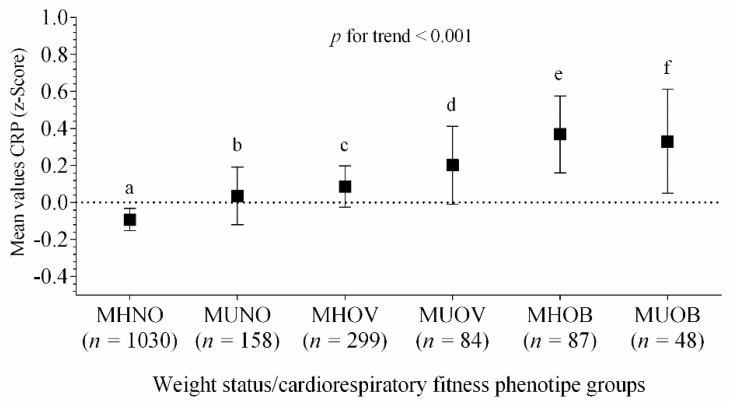
Differences in the means values of high-sensitive CRP Z-score across metabolic phenotypes of weight status. Bars represent adjusted means and 95% confidence intervals, for age, sex, pubertal stage and country, as confounders. The dashed line represents a value of zero for the scores, and a higher score represents greater high-sensitive CRP levels. Differences (≠) between groups, after Bonferroni adjustment, a ≠ (c, d, e, f); b ≠ (e, f); c ≠ (a, e); (*p* < 0.04 for all). Metabolically healthy non-overweight (MHNO); metabolically unhealthy non-overweight (MUNO); metabolically healthy overweight (MHOV); metabolically unhealthy overweight (MUOV); metabolically healthy obese (MHOB); and metabolically unhealthy obese (MUOB).

Figure 2 shows the differences in the high-sensitive CRP through all the groups, according to their metabolic phenotypes of weight status. Higher levels of CRP Z-score were associated for MHOV, MUOV, MHOB and MUOB groups (*p* for trend <0.001), after adjustments for age, sex, pubertal stage and country. 

**Figure 3:**
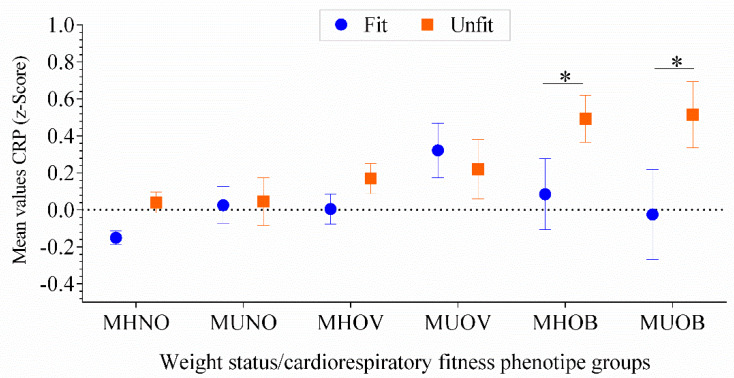
High-sensitive CRP levels across different metabolic groups. Two-way ANCOVA models adjusted for age, sex, pubertal stage and country (F = 4.423, *p* = 0.03). The dashed line represents a value of zero for the scores, and a higher score represents a greater high-sensitive CRP Z-score. The asterisks show the difference in the high-sensitive CRP z-scores for the unfit adolescents compared with the fit adolescents (*p* < 0.04 for all). MHNO, *n* = 720 (fit) and 310 (unfit); MUNO, *n* = 99 (fit) and 59 (unfit); MHOV, *n* = 151 (fit) and 148 (unfit); MUOV, *n* = 46 (fit) and 38 (unfit); MHOB *n* = 27 (fit) and 60 (unfit). MUOB *n* = 17 (fit) and 31 (unfit). Metabolically healthy non-overweight (MHNO); metabolically unhealthy non-overweight (MUNO); metabolically healthy overweight (MHOV); metabolically unhealthy overweight (MUOV); metabolically healthy obese (MHOB); and metabolically unhealthy obese (MUOB).

Figure 3 displays mean values of high-sensitive CRP Z-score in each of the metabolic health phenotypes in low- and high-CRF adolescents. There was a trend for high-sensitive CRP Z-score through each metabolic health phenotypes, with the CRF attenuating the association of high-sensitive CRP Z-score levels, particularly in the MHNO and in adolescents with obesity, regardless of their metabolic profile (F = 4.423, *p* = 0.03). 

## 4. Discussion

Our study showed that high-sensitive CRP levels elevated linearly across weight status, without significant differences between metabolic phenotypes. Furthermore, these results also suggest that high levels of CRF attenuates the association of CRP levels, particularly with participants within the MHNO, MHOB and MUOB categories. These results add to the findings of the existing literature about the relevance of CRF on inflammation throughout weight status and metabolic phenotypes categories. Moreover, our findings support previous studies which demonstrated that obesity and the metabolic disorders’ risk in children and adolescents are associated with low-grade inflammation, especially on CRP [32,33]. Indeed, CRP has been directly associated with insulin measures, triglyceride, inversely with HDL-c [34] and a metabolic risk score [6].

Some studies have shown that MHOB subjects with low CRP levels had a trend toward lowering CVD risk than those MHOB with high levels of CRP, along with a similar risk to that subjects with healthy profile and non-obese [35]. Our findings also showed significant differences throughout weight status, but not for metabolic phenotypes in the same weight status category. These results are in agreement with a recent study that showed that adolescents with higher values of C-reactive protein had a higher probability of being in the MHO (obese/overweight) group than those from other categories [36].

Several epidemiology studies have widely adopted BMI as a marker of adiposity; however, this measure has been questioned by some authors who see it as being inaccurate [37]. Nevertheless, our results are in accordance with previous findings, showing that high levels of CRF are associated with systemic inflammation, regardless of weight status [38,39,40]. It seems that CRF per se also plays an important role by inducing an anti-inflammatory status, namely on increased systemic levels of some types of cytokines with anti-inflammatory properties [41]. In addition, adolescents with obesity and high levels of CRF may have other fat deposits, besides the excessive fat, that contribute to a low-grade inflammation [42]. The chronic-inflammation-related co-morbidities may contribute to disability and decreased CRF level, inducing accumulation of excessive fat and thereby enhancing inflammation in a positive feedback loop [42]. 

Future studies are needed in order to confirm the extent that weight status and CRF remains at cardiometabolic disease risk compared with counterparts with no excess weight. For example, exercise interventions in youth have shown to improve CRF on average by 30%, including among children and adolescents with overweight and obesity [43]. Our results also have public health and clinical implications, as currently, obesity treatment is focused largely on energy restriction; however, we observed that CRF can attenuate the detrimental association of inflammation, especially in obese adolescents, independently of health or unhealthy phenotype. 

The current study is subject to certain limitations. First, the cross-sectional design of the study made it impossible to draw any causal inferences. Furthermore, blood samples only reflect inflammatory status at one time point. Additional biomarkers, such as fibrinogen, leptin, adiponectin, interleukin-6, interferon alpha and complementary factors could help to address a more complete inflammatory profile. Finally, the current results should be interpreted with caution, since no direct measure of absolute CRF was available in our study.

This study has several strengths. The first strength is the novelty of analyzing the data according with the metabolic profile in adolescents. In addition, the relatively large sample size, particularly with data derived from blood samples, is also a strength.

## 5. Conclusions

In summary, this large analysis suggests that CRF is a potential mechanism underlying the associations between weight status, metabolic profile and inflammation. Additional longitudinal analyses shall be conducted to further investigate the causal relationship between these variables. Finally, the role of other lifestyles determinants—in particular, sleep and dietary intake—shall further be investigated as potential confounders of the associations between CRF, weight status, metabolic profile and inflammation.

## Figures and Tables

**Table 1:** Participants’ characteristics, according to the metabolic health phenotype.

Characteristics	Non-Overweight (*n* = 1188)		Overweight (*n* = 383)		Obese (*n* = 135)	
	Metabolically Healthy (MHNO) (*n* = 1030)	Metabolically Unhealthy (MUNO) (*n* = 158)	Metabolically Healthy (MHOV) (*n* = 299)	Metabolically Unhealthy (MUOV) (*n* = 84)	Metabolically Healthy (MHOB) (*n* = 87)	Metabolically Unhealthy (MUOB) (*n* = 48)
Age (year)	14.9 (1.7)	14.9 (1.6)	14.8 (1.7)	14.5 (1.6)	14.6 (1.8)	14.7 (1.8)
Weight (kg)	50.6 (9.1)	53.8 (9.9)	60 (9.3)	66.7 (12)	76.1 (13)	78.8 (15.1)
Height (cm)	161 (09)	164 (09)	158 (08)	163 (08)	160 (09)	162 (10)
Body mass index	19.6 (2.0)	19.9 (1.9)	23.7 (1.9)	24.7 (1.9)	29.1 (3.1)	30.2 (3.2)
Triglycerides (mg/dL)	69.1 (27.1)	102.5 (53.1)	69.2 (24.2)	111.5 (58.9)	78.4 (41.9)	117.6 (63.8)
Glucose (mg/dL)	85.7 (13)	91.7 (20)	85.7 (11.8)	87.7 (17)	88.3 (9.1)	90.3 (13.1)
Systolic blood pressure (mmHg)	112.6 (12.6)	122.3 (12.8)	115.8 (11.5)	124.7 (13.1)	121.0 (13.6)	131.1(12.5)
Diastolic blood pressure (mmHg)	65.4 (8.6)	72.4 (9.6)	66.0 (8.4)	72.5 (8.9)	66.7 (8.9)	73.0 (9.2)
HDL-Cholesterol (mg/dL)	54.7 (12.3)	42.7 (10.5)	53.1 (12.3)	41.5 (7.9)	47.8 (8.5)	42.0 (8.5)
high-sensitive CRP (mg/L)	0.7 (0.07)	1.7 (0.2)	1.1 (0.15)	1.5 (0.2)	1.5 (0.2)	1.4 (0.2)
CRF VO_2_ peak (mL/kg/min)	42.07 (6.3)	40.1 (6.1)	38.6 (6)	37.9 (5.6)	36.6 (5.2)	35.7 (5.1)
CRF Number of Laps	50.4 (24.1)	42.5 (23.6)	39.5 (21.6)	31.7 (18.2)	27.7(22.4)	24.7 (15)
Pubertal stages						
Pubic hair development: ≤III/IV/V (%)	Girls 55.4/34.3/10.3		Boys 53.2/37.3/9.5		Total 54.3/35.8/9.9	
Breast–genital development: ≤III/IV/V (%)	57.1/37.3/5.6		59.7/31.1/9.2		58.3/34.4/7.3	

Data are presented as mean ± SD or number (percentage) of participants.
